# Supplementary material for: Deletion of Stk11 and Fos in mouse BLA projection neurons alters intrinsic excitability and impairs formation of long-term aversive memory
Source: eLife. 2020 Aug 11;9:e61036. doi: 10.7554/eLife.61036 (PMC7445010; doi:10.7554/eLife.61036)
Supplement: Figure 1—source data 1. — This data relates to Figure 1 panel B. [file elife-61036-fig1-data1.docx]

|  | Saline |  |  | Actinomycin D | |
| --- | --- | --- | --- | --- | --- |
|  | CTA_training | CTA_test |  | CTA_training | CTA_test |
| 1 | 0.8 | 0.1 | 1 | 0.9 | 0.7 |
| 2 | 0.9 | 0.2 | 2 | 1.2 | 0.4 |
| 3 | 1.1 | 0.2 | 3 | 0.6 | 0.7 |
| 4 | 0.9 | 0.5 | 4 | 0.6 | 0.4 |
| 5 | 1.2 | 0.1 | 5 | 0.9 | 1.2 |
| 6 | 0.9 | 0.2 | 6 | 1.3 | 0.7 |

**Figure 1-Source data 1.** Saccharin consumption (ml) during CTA training and test. This data relates to Figure 1 panel B.
