# Supplementary material for: Deletion of Stk11 and Fos in mouse BLA projection neurons alters intrinsic excitability and impairs formation of long-term aversive memory
Source: eLife. 2020 Aug 11;9:e61036. doi: 10.7554/eLife.61036 (PMC7445010; doi:10.7554/eLife.61036)
Supplement: Figure 1—source data 2. — This data relates to Figure 1 panel C. [file elife-61036-fig1-data2.docx]

|  | Saline |  | Actinomycin D |
| --- | --- | --- | --- |
|  | Fraction  consumed | | Fraction consumed |
|  | (test/training) |  | (test/training) |
| 1 | 0.125 | 1 | 0.777777778 |
| 2 | 0.222222222 | 2 | 0.333333333 |
| 3 | 0.181818182 | 3 | 1.166666667 |
| 4 | 0.555555556 | 4 | 0.666666667 |
| 5 | 0.083333333 | 5 | 1.333333333 |
| 6 | 0.222222222 | 6 | 0.538461538 |

**Figure 1-Source data 2.** Fraction of saccharin consumed (Test/Training). This data relates to Figure 1 panel C.
