# Supplementary material for: Deletion of Stk11 and Fos in mouse BLA projection neurons alters intrinsic excitability and impairs formation of long-term aversive memory
Source: eLife. 2020 Aug 11;9:e61036. doi: 10.7554/eLife.61036 (PMC7445010; doi:10.7554/eLife.61036)
Supplement: Figure 2—source data 1. — This data relates to Figure 2, panel C. [file elife-61036-fig2-data1.docx]

| Fos mRNA expression in YFPH+ neurons | | | | Stk11 mRNA expression in YFPH+ neurons | | | |
| --- | --- | --- | --- | --- | --- | --- | --- |
|  | CTA |  | Control |  | CTA |  | Control |
| 1 | 590.6706 | 1 | 119.1335 | 1 | 47.74758 | 1 | 71.272515 |
| 2 | 679.0608 | 2 | 2.566835 | 2 | 59.67813 | 2 | 66.143336 |
| 3 | 473.2317 | 3 | 2.990958 | 3 | 59.08793 | 3 | 137.85988 |
| 4 | 100.2304 | 4 | 275.3087 | 4 | 46.60252 | 4 | 124.72427 |

**Figure 2-Source data 1.** qPCR validation for Fos and Stk11 mRNA expression in YFPH+ neurons. This data relates to Figure 2, panel C.
