# Supplementary material for: Deletion of Stk11 and Fos in mouse BLA projection neurons alters intrinsic excitability and impairs formation of long-term aversive memory
Source: eLife. 2020 Aug 11;9:e61036. doi: 10.7554/eLife.61036 (PMC7445010; doi:10.7554/eLife.61036)
Supplement: Figure 2—figure supplement 1—source data 1. — This data relates Figure 2—figure supplement 1 panel B. [file elife-61036-fig2-figsupp1-data1.docx]

|  | Fos protein counts  in BLA | | |
| --- | --- | --- | --- |
|  | CTA |  | LiCl Control |
| 1 | 38 | 1 | 8 |
| 2 | 26 | 2 | 11 |
| 3 | 15 | 3 | 8 |
| 4 | 32 | 4 | 0 |

**Figure 2-Figure supplement 1-Source data 1.** FOS protein counts in BLA 4 hours following CTA training and LiCl control. This data relates Figure 2-Figure supplement 1 panel B.
