# Supplementary material for: Deletion of Stk11 and Fos in mouse BLA projection neurons alters intrinsic excitability and impairs formation of long-term aversive memory
Source: eLife. 2020 Aug 11;9:e61036. doi: 10.7554/eLife.61036 (PMC7445010; doi:10.7554/eLife.61036)
Supplement: Figure 2—figure supplement 2—source data 1. — This data relates Figure 2—figure supplement 2. [file elife-61036-fig2-figsupp2-data1.docx]

|  | CTA |  | Control |
| --- | --- | --- | --- |
| 1 | 253.4697 | 1 | 85.33251 |
| 2 | 349.8284 | 2 | 125.0663 |
| 3 | 163.5381 | 3 | 89.60124 |
| 4 | 228.7906 | 4 | 132.1286 |
| 5 | 69.1447 | 5 | 22.60647 |
| 6 | 80.44415 | 6 | 145.2649 |
| 7 | 132.1266 | 7 | 100 |
| 8 | 111.1281 | 8 | 100 |
| 9 | 242.0937 |  |  |

**Figure 2-Figure supplement 2-Source data 1.** STK11 protein levels in the BLA 4 hours following CTA training or taste control. This data relates Figure 2-Figure supplement 2.
