# Supplementary material for: Deletion of Stk11 and Fos in mouse BLA projection neurons alters intrinsic excitability and impairs formation of long-term aversive memory
Source: eLife. 2020 Aug 11;9:e61036. doi: 10.7554/eLife.61036 (PMC7445010; doi:10.7554/eLife.61036)
Supplement: Figure 3—source data 2. — This data relates to Figure 3 panel D. [file elife-61036-fig3-data2.docx]

|  | GFP injected |  | Cre injected |
| --- | --- | --- | --- |
|  | Fraction consumed |  | Fraction consumed |
|  | (test/training) |  | (test/training) |
| 1 | 0.230769231 | 1 | 0.714285714 |
| 2 | 0.333333333 | 2 | 0.222222222 |
| 3 | 0.363636364 | 3 | 0.571428571 |
| 4 | 0.1875 | 4 | 0.583333333 |
| 5 | 0.272727273 | 5 | 0.1 |
| 6 | 0.333333333 | 6 | 0.461538462 |
| 7 | 0 | 7 | 0.692307692 |
| 8 | 0.333333333 | 8 | 0.583333333 |

**Figure 3-Source data 2.** Fraction of saccharin consumed (Test/Training).

This data relates to Figure 3 panel D.
