# Supplementary material for: Deletion of Stk11 and Fos in mouse BLA projection neurons alters intrinsic excitability and impairs formation of long-term aversive memory
Source: eLife. 2020 Aug 11;9:e61036. doi: 10.7554/eLife.61036 (PMC7445010; doi:10.7554/eLife.61036)
Supplement: Figure 4—source data 2. — This data relates to Figure 4 panel B. [file elife-61036-fig4-data2.docx]

|  | GFP injected |  | Cre injected |
| --- | --- | --- | --- |
|  | Fraction  consumed | | Fraction  consumed |
|  | (test/training) |  | (test/training) |
| 1 | 0.25 | 1 | 0.818181818 |
| 2 | 0.5 | 2 | 0.75 |
| 3 | 0.090909091 | 3 | 1 |
| 4 | 0.5 | 4 | 0.9 |
| 5 | 0.375 | 5 | 0.8 |
| 6 | 0.285714286 | 6 | 0.8 |
|  |  | 7 | 0.444444444 |

**Figure 4-Source data 2.** Fraction of saccharin consumed (Test/Training). This data relates to Figure 4 panel B.
