# Supplementary material for: Deletion of Stk11 and Fos in mouse BLA projection neurons alters intrinsic excitability and impairs formation of long-term aversive memory
Source: eLife. 2020 Aug 11;9:e61036. doi: 10.7554/eLife.61036 (PMC7445010; doi:10.7554/eLife.61036)
Supplement: Figure 4—source data 4. — This data relates to Figure 4 panel E. [file elife-61036-fig4-data4.docx]

|  | GFP injected |  | Cre injected |
| --- | --- | --- | --- |
|  | Fraction  consumed | | Fraction  consumed |
|  | (test/training) |  | (test/training) |
| 1 | 0.166666667 | 1 | 0.428571429 |
| 2 | 0.6 | 2 | 0.142857143 |
| 3 | 0.25 | 3 | 0.714285714 |
| 4 | 0.833333333 | 4 | 0.166666667 |
| 5 | 0.333333333 | 5 | 0.333333333 |
| 6 | 0.125 | 6 | 0.166666667 |
| 7 | 0.142857143 |  |  |

**Figure 4-Source data 4.** Fraction of saccharin consumed (Test/Training). This data relates to Figure 4 panel E.
