# Supplementary material for: Deletion of Stk11 and Fos in mouse BLA projection neurons alters intrinsic excitability and impairs formation of long-term aversive memory
Source: eLife. 2020 Aug 11;9:e61036. doi: 10.7554/eLife.61036 (PMC7445010; doi:10.7554/eLife.61036)
Supplement: Figure 5—figure supplement 1—source data 1. — This data relates to Figure 5—figure supplement 1 panel A. [file elife-61036-fig5-figsupp1-data1.docx]

|  | Stk11 f/f mice | |
| --- | --- | --- |
|  | GFP | Cre |
| 1 | 138.5198 | 179.016 |
| 2 | 78.21699 | 87.04794 |
| 3 | 83.26325 | 81.3709 |

**Figure 5-Figure supplement 1-Source data 1.** *Stk11* deletion in BLApn does not change C-FOS expression. This data relates to Figure 5-Figure supplement 1 panel A.
