# Supplementary material for: Deletion of Stk11 and Fos in mouse BLA projection neurons alters intrinsic excitability and impairs formation of long-term aversive memory
Source: eLife. 2020 Aug 11;9:e61036. doi: 10.7554/eLife.61036 (PMC7445010; doi:10.7554/eLife.61036)
Supplement: Figure 5—figure supplement 1—source data 2. — This data relates to Figure 5—figure supplement 1 panel B. [file elife-61036-fig5-figsupp1-data2.docx]

|  | Fos f/f mice | |
| --- | --- | --- |
|  | GFP | Cre |
| 1 | 77.65713 | 40.98068 |
| 2 | 169.5169 | 161.3579 |
| 3 | 52.826 | 101.8156 |

**Figure 5-Figure supplement 1-Source data 2.** *Fos* deletion in BLApn does not change C-FOS expression. This data relates to Figure 5-Figure supplement 1 panel B.
