# Supplementary material for: Deletion of Stk11 and Fos in mouse BLA projection neurons alters intrinsic excitability and impairs formation of long-term aversive memory
Source: eLife. 2020 Aug 11;9:e61036. doi: 10.7554/eLife.61036 (PMC7445010; doi:10.7554/eLife.61036)
Supplement: Figure 8—source data 2. — This data relates to Figure 8 panel B. [file elife-61036-fig8-data2.docx]

|  | GFP | hM3Dq |
| --- | --- | --- |
| 1 | 0.454545 | 0.6 |
| 2 | 0.083333 | 0.5 |
| 3 | 0.071429 | 1 |
| 4 | 0.166667 | 0.444444 |
| 5 | 0 | 0.833333 |
| 6 | 0 | 0.916667 |
| 7 | 0.428571 | 1.2 |
| 8 | 0.666667 | 0.75 |
| 9 | 0.571429 | 0.333333 |

**Figure 8-Source data 2.** Fraction of saccharin consumed (Test/Training). This data relates to Figure 8 panel B.
